# Advanced Management of Patients Undergoing Transcatheter Treatment for Pulmonary Embolism: Evidence-Based Strategies for Optimized Patient Care

**DOI:** 10.3390/jcm13247780

**Published:** 2024-12-20

**Authors:** Francesco Costa, Alfonso Jurado-Román, Gabriele Carciotto, Victor Becerra-Munoz, Daniel Tébar Márquez, Felix Götzinger, Enrico Cerrato, Shantum Misra, Marco Spissu, Marco Pavani, Marco Mennuni, Fernando Carrasco Chinchilla, Antonio Dominguez-Franco, Antonio Muñoz-Garcia, Rocio Sanchez Navarrete, Ferdinando Varbella, Pablo Salinas-Sanguino, Eric A. Secemsky, Felix Mahfoud, Antonio Micari, Juan Horacio Alonso-Briales, Manuel Jimenez Navarro

**Affiliations:** 1 Área del Corazón, Instituto de Investigación Biomédica de Málaga y Plataforma en Nanomedicina (IBIMA Plataforma BIONAND), Hospital Universitario Virgen de la Victoria, Centro de Investigación Biomédica en Red de Enfermedades Cardiovasculares (CIBERCV), Departamento de Medicina UMA, 29010 Malaga, Spain; dottfrancescocosta@gmail.com (F.C.); vmbecerram@gmail.com (V.B.-M.); fernandocarrascochinchilla@gmail.com (F.C.C.); adominguez74@gmail.com (A.D.-F.); ajmunozgarcia@secardiologia.es (A.M.-G.); rochupry@hotmail.com (R.S.N.); juanhalonso@secardiologia.es (J.H.A.-B.); 2 Department of Biomedical and Dental Sciences and of Morphological and Functional Images, University of Messina, 98122 Messina, Italy; gcarciotto97@gmail.com (G.C.); micariantonio@gmail.com (A.M.); 3 Cardiology Department, University Hospital La Paz, 28046 Madrid, Spain; alfonsojuradoroman@gmail.com (A.J.-R.); daniel.tebar.m@gmail.com (D.T.M.); 4 Department of Internal Medicine III—Cardiology, Angiology and Intensive Care Medicine, Saarland University Hospital Homburg, Saarland University, 66123 Saarbrücken, Germany; felix.goetzinger@uks.eu (F.G.); felix.mahfoud@gmail.com (F.M.); 5 Interventional Cardiology Unit, San Luigi Gonzaga University Hospital, Orbassano and Rivoli Infermi Hospital, 10098 Turin, Italy; enrico.cerrato@gmail.com (E.C.); marco.spissu10@gmail.com (M.S.); marcopavani@alice.it (M.P.); ferdinando.varbella@aslto3.piemonte.it (F.V.); 6 Beth Israel Deaconess Medical Center, Harvard Medical School, Boston, MA 02215, USA; smisra1@bidmc.harvard.edu (S.M.); ericsecemsky@gmail.com (E.A.S.); 7 Richard A. and Susan F. Smith Center for Outcomes Research in Cardiology, Boston, MA 02215, USA; 8 Division of Cardiology, Maggiore della Carità Hospital, 28100 Novara, Italy; marco.mennuni@gmail.com; 9 Servicio de Cardiología, Hospital Clínico San Carlos, 28040 Madrid, Spain; salinas.pablo@gmail.com

**Keywords:** pulmonary embolism, catheter-directed thrombolysis, catheter-directed mechanical thrombectomy, pulmonary hypertension

## Abstract

Pulmonary embolism (PE) is a life-threatening medical condition caused by the thrombotic occlusion of one or more branches of the lung vasculature, which represents the third most common cause of cardiovascular mortality after myocardial infarction and stroke. PE treatment requires a tailored approach based on accurate risk stratification and personalized treatment decision-making. Anticoagulation is the cornerstone of PE management, yet patients at higher clinical risk may require more rapid reperfusion therapies. In recent years, transcatheter treatment has emerged as a valuable option for patients with intermediate–high or high-risk PE who have contraindications to systemic thrombolysis. Recent advancements in catheter-directed therapies, such as catheter-directed thrombolysis (CDT) and catheter-directed mechanical thrombectomy (CDMT), provide minimally invasive options for swift symptom relief and hemodynamic stabilization. This review aims to provide a practical approach for optimal patient selection and management for PE percutaneous therapies, supported by a thorough evaluation of the current evidence base supporting these procedures. A focus on post-procedural management, the prevention of recurrence, and monitoring for long-term complications such as chronic pulmonary hypertension and post-PE syndrome is also specifically tackled.

## 1. Introduction

Pulmonary embolism (PE), consisting of the occlusion of one or more branches of lung vasculature, usually caused by emboli originating from venous thrombi [1], is the third most frequent cause of cardiovascular (CV) death and represents a major cause of morbidity and mortality worldwide [2]. From a clinical standpoint, it represents a challenge for clinicians, owing to its diverse clinical presentation and poor outcomes [3]. In addition, patients who survive after an acute PE may suffer considerable long-term complications impacting functional status, quality of life, and prognosis [4,5]. While anticoagulation represents the treatment of choice in the majority of patients [6], reperfusion strategies such as systemic thrombolysis or invasive strategies, either surgical or catheter-directed, may be needed in higher-risk patients [7]. Systemic thrombolysis is recommended as the first-line treatment in hemodynamically unstable patients but is underutilized due to a perceived increased risk of bleeding [8,9]. In recent years, the development of catheter-directed therapies has expanded treatment options for patients with intermediate–high and high-risk PE, offering targeted thrombus removal with a potentially lower risk of systemic complications. This review aims to provide an overview of current patient selection for PE percutaneous treatment and a comprehensive outline of the current and upcoming evidence supporting these procedures. It will also explore the role of follow-up care, including the management of pulmonary hypertension, a rare but dreadful complication of PE.

## 2. Risk Stratification and Patient Selection

**Figure 1:**
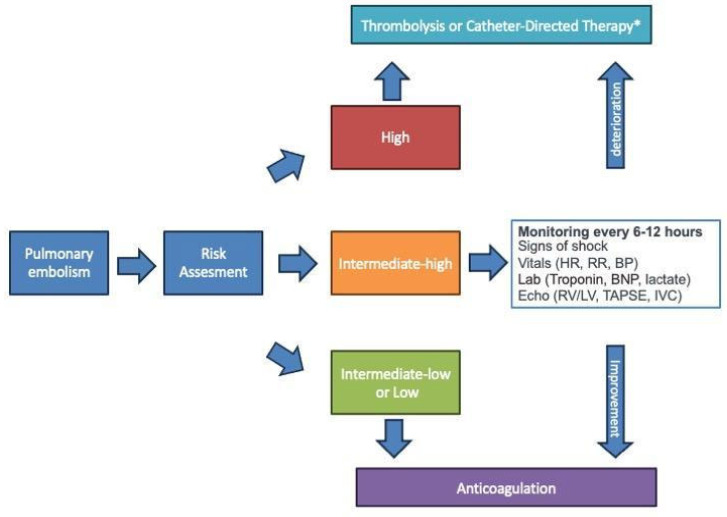
Decision-making algorithm from diagnosis to treatment of pulmonary embolism. * if thrombolysis has failed (judged by persistent clinical instability and unchanged RV dysfunction on echocardiography after 36 h) or is contraindicated (absolute contraindications to fibrinolysis: history of hemorrhagic stroke or stroke of unknown origin; ischemic stroke in previous 6 months; central nervous system neoplasm, major trauma, surgery, or head injury in previous 3 weeks; bleeding diathesis; active bleeding. Relative contraindications to fibrinolysis: transient ischemic attack in previous 6 months, oral anticoagulation, pregnancy or first post-partum week, non-compressible puncture sites, traumatic resuscitation, refractory hypertension (systolic BP > 180 mmHg), advanced liver disease, infective endocarditis, active peptic ulcer) [5]. Legend: BNP = B natriuretic peptide, BP = blood pressure, LV = left ventricle, HR = heart rate, IVC = inferior vena cava, RR = respiratory rate, RV = right ventricle, TAPSE = tricuspid annular plane systolic excursion.

Once PE is diagnosed, accurate risk stratification is key for optimal treatment selection. PE severity and associated early mortality risk are stratified using clinical, laboratory, and imaging parameters. While low- and intermediate–low-risk patients can be safely treated with oral anticoagulation in a low-intensity monitoring environment or at home, higher therapeutic efforts should be focused on those patients at intermediate–high or high risk (Figure 1). High-risk patients are characterized by hemodynamic instability, which may include symptoms such as sustained hypotension (systolic blood pressure less than 90 mmHg or a drop of more than 40 mmHg for at least 15 min not attributable to other causes), shock, or signs of end-organ dysfunction indicative of poor perfusion [5]. This presentation is associated with a significant risk of early mortality (up to 27% within 24 h and 49% within 72 h) [10] and necessitates prompt reperfusion therapy to stabilize hemodynamic and gas exchange (Figure 1). While there has been a noticeable rise in the use of catheter-based therapies for acute PE, a significant shift toward a more interventional strategy for high-risk PE has yet to occur. Currently, primary reperfusion using CDT is not the first-line treatment for patients with high-risk acute PE, nor for any of the other PE risk categories. Instead, according to current guidelines, CDT should be considered for patients with high-risk PE in whom thrombolysis is contraindicated or has failed [5,7,11,12]. CDT should also be considered a rescue treatment for initially stable patients in whom anticoagulant treatment fails, i.e., those who experience hemodynamic deterioration [5]. Notably, the available data on high-risk patients are minimal, rendering all the above recommendations weak with low levels of evidence. Intermediate–high-risk PE patients carry a 5–10% mortality risk at 30 days [13] and are characterized by signs of right ventricular dysfunction (RVD) on echocardiography or computed tomography (CT) pulmonary angiography, combined with elevated cardiac biomarkers indicating myocardial injury. The mainstay treatment in these patients is anticoagulation, whereas routine systemic thrombolysis is contraindicated [5,14]. Nonetheless, the specific goal in this group is the early detection of clinical deterioration and prevention of progression to high-risk status and hemodynamic instability for eventual treatment escalation. Early identification of clinical deterioration is complex, and numerous clinical (e.g., heart rate, respiratory rate, blood pressure, saturation) [15], imaging (e.g., right ventricle/left ventricle (RV/LV) ratio, tricuspid annular plane systolic excursion (TAPSE), inferior vena cava dilatation) [16], and laboratory (e.g., troponin, brain natriuretic peptide, lactates) [17] prognostic indicators have been proposed. Close monitoring within 6–12 h without improvement or with evidence of progressive heart rate escalation despite anticoagulation can guide the selection of timely reperfusion therapy. In this setting, transcatheter treatment should be considered, particularly if high-bleeding risk or contraindications to thrombolysis limit the therapeutic opportunities. Conditions at increased risk of complications from the transcatheter approach should also be factored in for decision-making for treatment selection, such as coagulation disorders, previous allergic reactions to iodinated contrast medium, or the presence of advanced kidney diseases. Finally, a global risk–benefit assessment is critical in patients with limited life expectancy or with lower cardiac or pulmonary reserves where treatment benefits could be minimal due to the marginal impact of the acute PE on patient condition and limited possibilities of functional recovery [18]. For this reason, establishing a pulmonary embolism response team (PERT) where treatment decisions are shared by a panel of different specialists, including cardiologists, interventional cardiologists, cardiothoracic surgeons, anesthesiologists, radiologists, and pulmonologists, could play a crucial role in the management of acute PE, particularly in complex or high-risk cases, granting a rapid and coordinated approach to assessment and treatment. PERTs facilitate timely decision-making by integrating expertise to evaluate the severity of PE, stratify risk, and select the most appropriate therapeutic strategy. Their collaborative model not only optimizes patient outcomes by ensuring individualized care but also reduces mortality and long-term complications associated with delayed or suboptimal treatment [19,20,21].

## 3. Current Evidence Supporting Percutaneous Treatment of Pulmonary Embolism

### 3.1. Evidence for Catheter Directed Thrombolysis

In CDT, as said before, a low-dose thrombolytic agent is delivered by a catheter directly into the pulmonary artery or into the thrombus, thereby reducing the total dose of the thrombolytic and possibly reducing bleeding complications. Thrombolytic infusion could be performed either with standard catheters or via dedicated catheters [22,23].

The open-label randomized ULTIMA trial compared ultrasound-assisted CDT via an EKOS device in 59 patients at intermediate-high-risk PE, which provides ultrasound-accelerated catheter-directed thrombolysis combining local infusion of the fibrinolytic agent with the emission of 2 MHz ultrasound waves to enhance fibrin fiber disaggregation, with the standard of care or parenteral anticoagulation. The experimental treatment was based on the infusion of a low-dose recombinant tissue plasminogen activator (TPA) (10–20 mg) over 15 h, while standard anticoagulation was performed with heparin alone. The results showed a greater reduction in the RV/LV ratio at 24 h with CDT (0.30 ± 0.20 in the CDT group compared with 0.03 ± 0.16 in the control group; *p* < 0.001). Mean PAP was reduced by 5.7 ± 7.6 mmHg within 12 h. Only three minor bleedings were registered in the CDT group compared with one minor bleeding event in the control group (*p* = 0.61). However, the sample size was small and the trial was not powered for hard clinical-end points [24]. These results were confirmed in the larger SEATTLE II study, a prospective, single-arm, multicenter study that evaluated the safety and efficacy of USCDT using the EKOS system. As in ULTIMA, the TPA regimen used high dosages and long infusion times (1 mg/h for 24 h for predominantly unilateral PE or 1 mg/h for 12 h for bilateral PE). The study showed a 25% decrease in CT-measured RV/LV diameter ratio over 48 h, and a 30% decrease in pulmonary artery systolic pressure (PAPs). A total of 15 (10%) instances of Global Utilization of Streptokinase and Tissue plasminogen activator for Occluded arteries (GUSTO) moderate or severe bleeding were observed [25].

The open-label randomized SUNSET PE (standard vs. ultrasound-assisted catheter thrombolysis for submassive Pulmonary Embolism) trial compared the effectiveness of ultrasound catheter-directed thrombolysis (USCDT) (using the EKOS System) with standard CDT (using the Uni-Fuse or Cragg–McNamara systems). The study objective was to evaluate the reduction of thrombus burden after treatment in 82 patients with acute intermediate–high-risk PE. The study reported no significant differences in thrombus reduction with the refined Miller score between standard CDT and USCDT using comparable lytic doses. The mean RV-to-LV ratio was more markedly decreased in the CDT group than in the USCDT group (0.59 ± 0.42 versus 0.37 ± 0.34; *p* = 0.01). Additionally, two major bleeding events, including one hemorrhagic stroke and three minor bleeding events, occurred after USCDT, whereas no bleeding complications occurred in the CDT group. However, the interpretation of these results should be in light of several limitations, including the small sample size and the lack of standardization of the thrombolytic regimens [26].

The CANARY trial was an open-label randomized study that evaluated the effects of CDT (alteplase, 0.5 mg/catheter/h for 24 h) combined with heparin against anticoagulation alone in patients with acute intermediate–high-risk pulmonary embolism. The study was prematurely stopped owing to the emergence of the COVID-19 pandemic, hence not providing definitive conclusions. After the inclusion of 93 patients, no statistically significant difference in the rate of the primary outcome (the proportion of patients with a 3-month echocardiographic RV/LV ratio greater than 0.9, assessed by a core laboratory) was observed between the two study arms. Nevertheless, a numerical reduction of the primary outcome was observed in the CDT group, with 4.3% of patients in the CDT group versus 12.8% in the anticoagulation group reaching the study primary endpoint. In addition, at three months, CDT patients had a significantly lower median RV/LV ratio (0.7 IQR 0.6–0.7 vs. 0.8 IQR 0.7–0.9 *p* = 0.01), and fewer CDT patients experienced the combined outcome of death or an RV/LV ratio over 0.9 (4.3% vs. 17.3%; OR 0.20; 95% CI 0.04–1.03; *p* = 0.048). Only one nonfatal major gastrointestinal bleeding event was reported in the CDT group [27].

The thrombolytic dose and duration strategies were evaluated in the OPTALYSE PE study, a randomized, multicenter, and parallel-group trial. A total of 101 patients were randomized to receive one of four different regimens testing the efficacy and safety of lower doses over shorter treatment durations compared to previous studies: 4 mg/lung/2 h, 4 mg/lung/4 h, 6 mg/lung/6 h, and 12 mg/lung/6 h during treatment with USCDT. RV/LV diameter ratio by angio-CT at 48 h, the study’s primary endpoint, improved in each of the four arms by approximately 25%. From a safety point of view, the study was not powered to detect statistically significant differences between the study groups. Overall, five major instances of bleeding were observed in the first 72 h of treatment (zero, two, one, and two in the four study arms, from lower to higher doses, respectively) [28]. This concept has been recently confirmed in the prospective international registry KNOCKOUT-PE, which included 489 patients with intermediate–high or high-risk PE. In this study, the total mean r-TPA dose was 18.1 mg, with roughly 70% of patients treated with less than 20 mg and a mean duration of treatment of 10.5 h. Bleeding events (1.6%) and recurrent VTE (0.4%) were rare at 30 days with this approach [29].

Finally, a recent meta-analysis of eight observational studies involving 11,932 patients with high-risk or intermediate–high-risk PE compared the safety and efficacy of systemic thrombolysis and CDT. CDT was associated with significantly lower in-hospital mortality (risk ratio 0.52, 95% CI 0.40–0.68, *p* < 0.001) compared to systemic thrombolysis. No significant difference was found between the treatments in terms of major bleeding, except for intracranial hemorrhage, which was less frequent with CDT (RR 0.66, 95% CI 0.47–0.94, *p* = 0.02). However, most of the current evidence is based on observational studies and requires further support from large and adequately powered randomized trials [30].

### 3.2. Evidence for Catheter-Directed Mechanical Thrombectomy

Contemporary CDMT involves the percutaneous removal of thrombi from the pulmonary arteries using large- or medium-bore catheters attached to vacuum-generating devices. These procedures are commonly performed via femoral or jugular venous access under fluoroscopic guidance. The two most widely used devices are the Penumbra Indigo Thrombectomy System and the Inari FlowTriever System. The Penumbra Indigo System utilizes 12 or 16 French catheters connected to an electronic console, which manages aspiration force and provides continuous, controlled suction. The Inari FlowTriever System employs large-bore catheters ranging from 16 to 24 French, connected to a dedicated syringe for manual aspiration. This system also features self-expanding nitinol disks designed to engage and retrieve thrombus mechanically. More recently, the AngioDynamics AlphaVac F18 system has received regulatory approval. This device uses an 18 French large-bore angulated catheter with an expandable 33 French tip for thrombus engagement, connected to a manual aspiration system operated via an ergonomic handle. These devices have been studied in prospective studies, demonstrating their ability to reduce in-hospital RV/LV ratios compared to anticoagulation alone [31,32]. However, most evidence is based on single-arm studies and registries. Due to intrinsic limitations of observational data, meta-analyses in this space are also less reliable [33], and multicenter prospective studies are probably the best piece of evidence to date waiting for future RCTs (Table 1).

#### 3.2.1. Evidence for the Inari FlowTriever System

The FLARE study assessed the effectiveness and safety of the FlowTriever System for treating intermediate-risk PE. Conducted as a prospective, multicenter, single-arm trial, it included 106 patients diagnosed with intermediate-risk PE. The primary focus was on the reduction of the RV/LV ratio, evaluated through CT scans after the procedure. The results showed a significant 25% decrease in the RV/LV ratio and PAPs at 48 h after the procedure. Safety outcomes were favorable, occurring in 3.8% of included patients, with a low incidence of major bleeding and no cases of intracerebral hemorrhage [32].

The FLASH registry is an ongoing multicenter, prospective registry, evaluating the safety and efficacy of the FlowTriever device in patients at intermediate–high or high-risk PE. The results of the first 800 US patients enrolled (8% with high-risk PE, 92% with intermediate-risk PE) confirmed the efficacy of the FlowTriever system, with a post-procedural reduction of the RV-to-LV ratio (from 1.23 ± 0.36 to 0.98 ± 0.31; *p* < 0.0001) of PAP (from 32.6 ± 9.0 mmHg to 24.9 ± 8.9 mmHg; *p* < 0.0001).Three intraprocedural major adverse events were registered, two clinical deterioration and one tricuspid valve injury, and 11 major bleeding events occurred at 48 h after the procedure. At 30 days, the all-cause re-admission to hospital rate was 6.2%, and all-cause mortality was as low as 0.8% [34]. These data match with recent case series published in Europe [35].

The more recent FLAME study, a prospective, multicenter, observational study, focused on the impact of CDMT with FlowTriever exclusively in high-risk PE patients. The trial enrolled 53 patients in the FlowTriever Arm and 61 in the comparison group based on standard-of-care treatment with anticoagulation or thrombolysis according to physician preference. The results indicated that the primary endpoint, a composite of in-hospital all-cause mortality, bailout to an alternate thrombus removal strategy, clinical deterioration, or major bleeding occurred in 17.0% of patients treated with the FlowTriever and in 63.9% of those treated with standard of care. Specifically, in-hospital mortality rates were 1.9% for patients treated with the FlowTriever device compared to 29.5% for those managed with other methods [36]. This study, albeit not being randomized, highlights the large potential benefit of standardizing transcatheter treatment in patients with high-risk PE, a concept that should be explored in dedicated randomized trials.

#### 3.2.2. Evidence for the Penumbra Indigo/Lightning System

The EXTRACT-PE study is the first prospective, single-arm, multicenter study that assessed the safety and effectiveness of the Indigo Aspiration System in treating patients with symptomatic acute PE without hemodynamic instability. It included 119 patients with intermediate-risk PE. It showed notable enhancements in hemodynamic parameters, in particular, a 27% reduction in the RV/LV ratio, alongside a low incidence (1.7%) of major adverse events (two major bleedings and one pulmonary vascular injury). These findings, even with the limitations of a non-randomized trial and lacking a follow-up beyond 30 days, provided additional evidence in favor of aspiration embolectomy as a valid alternative to conventional anticoagulation therapy [31].

The STRIKE-PE, a prospective, multicenter, international study, will enroll 600 patients with acute PE and a RV-to-LV ratio of ≥0.9, who will receive endovascular treatment using the Indigo Aspiration System as a first-line therapy. The primary endpoints will assess changes in the RV/LV ratio and the occurrence of composite major adverse events (MAEs) within 48 h. The results from the first 150 enrolled patients (94.7% with intermediate-risk PE and 5.3% with high-risk PE) showed a mean decrease in systolic pulmonary artery pressure of 16.3% (*p* < 0.001) and a decrease in the RV/LV ratio from 1.39 to 1.01 at 48 h (25.7%; *p* < 0.001). These preliminary results also showed a composite MAE rate of 2.7% and significant clinical improvements in the patients based on QoL measures, Borg dyspnea scale, and NYHA class [37].

#### 3.2.3. Evidence for the Angiodynamics AlphaVac 18 System

The APEX-AV trial is a multicenter study evaluating the AlphaVac F1885 aspiration system for treating acute intermediate-risk PE. Eligible patients included those with CT-detected PE, a heart rate under 130 beats per minute, and an RV/LV ratio above 0.9. The primary efficacy outcome was the change in the RV/LV ratio at 48 h post-procedure, while primary safety concerns focused on MAEs. A total of 122 patients were treated and the results showed that the RV/LV ratio decreased by 0.45 and overall clot burden decreased by 35.5%. There were five MAEs, including five major bleeding incidents, but no deaths or thrombolytic treatments within 48 h. One patient had a symptomatic PE recurrence within 30 days. Overall, the AlphaVac F1885 system proved safe and effective, showing notable improvements in RV function and significant thrombus reduction [38].

#### 3.2.4. Evidence of CDMT vs. CDT

A recent analysis from the REAL-PE study, including 2259 real-world patients with acute PE treated with transcatheter techniques based on a large multicenter electronic health records system, compared USCDT with CDMT (USCDT = 1577—CDMT = 682). No differences in terms of in-hospital mortality or 30-day readmission were observed between the two transcatheter treatments. Major bleeding according to the International Society on Thrombosis and Hemostasis (ISTH) and the Bleeding Academic Research Consortium (BARC) definitions was more common after CDMT (ISTH: 17.3% vs. 12.4%—BARC 3b: 15.4% vs. 11.8%). Similarly, intracranial hemorrhage was more common after CDMT. After adjustment, CDMT remained an independent predictor of major bleeding [39]. Considering the observational design of this study and the significant risk of selection bias and residual confounding, these results should be considered exploratory while specific trials comparing USCDT and CDMT are in progress.

More recently, the PEERLESS study (NCT05111613), presented at the TCT 2024 conference, was the first multinational RCT comparing CDMT with the FlowTriever System vs. CDT. It randomized in a 1:1 fashion 550 patients with hemodynamically stable PE and RV dysfunction to the two transcatheter approaches. The primary endpoint, assessed at 7 days post-procedure, was a composite of mortality, bleeding, and duration of ICU stay. The study met its primary endpoint (95% CI: 3.68–6.97; *p* < 0.001), demonstrating that large-bore CDMT registered significantly fewer episodes of clinical deterioration and/or bailout escalation of treatment compared to CDT (1.8% vs. 5.4%; *p* = 0.04) and less postprocedural ICU admissions (41.6% vs. 98.6%) and stays >24 h (19.3% vs. 64.5%). In this randomized setting, there was no significant difference in mortality, intracranial hemorrhage, or major bleeding between strategies, and RV/LV ratio reduction and 30-day mortality were similar (0.32 ± 0.24 vs. 0.30 ± 0.26; *p* = 0.55; 0.4% vs. 0.8%; *p* = 0.62) [40].

**Table 1:** Multicenter prospective studies for catheter-directed mechanical thrombectomy.

Study Name	NCT	Year †	Patients	Risk Profile	Intervention	Control	Primary Outcome	Results
FLARE [32]	02692586	2019	106	Intermediate–High	FlowTriever	–	Change in RV/LV ratio per CTA at 48 h	>Reduction 48 h from baseline of 0.38, or 25.1% >3.8% MAE
EXTRACT PE [31]	03218566	2021	119	Intermediate–High	Indigo System (8Fr)	–	Change in RV/LV ratio per CTA at 48 h	>Reduction 48 h from baseline 0.43 (95%CI 0.38–0.47) 1.7% MAE
FLASH US ‡ [34]	03761173	2022	800	Any FT-treated PE (8% high-risk)	FlowTriever	–	Safety composite (48 h MAE §)	>14 (1.8%) MAE, including 11 major bleeds, two deaths >0.8% 30-day mortality
FLAME [36]	04795167	2023	114	High-risk	FlowTriever	Systemic thrombolysis or Anticoagulation (Performance Goal)	In-hospital efficacy composite *	>17% FlowTriever vs. 63.9% medical group (non-randomized comparison)
Sławek-Szmyt et al. ‡ [41]	04879069	2023	110	Any IS-treated PE (28.2% high-risk)	Indigo System (8Fr)	–	Safety composite (48 h MAE #)	>9% MAE including 5.5% death at 48 h, 1.8% pulmonary artery injury, 1.8% major bleeding
PEERLESS [39]	05111613	2024	550	Intermediate–High	FlowTriever	Catheter-directed thrombolysis	In-hospital efficacy composite **	Fewer episodes of clinical deterioration and/or bailout escalation of treatment (1.8% vs. 5.4), fewer postprocedural ICU admissions (41.6% vs. 98.6%) and stays >24 h (19.3% vs. 64.5%). No significant difference in mortality, intracranial hemorrhage, or major bleeding. Similar RV/LV ratio reduction and 30-day mortality

‡ Registry is ongoing. † Publication year. § Device-related death, major bleeding, device, or procedure-related adverse events. * All-cause mortality, bailout to an alternate thrombus removal strategy, clinical deterioration, and major bleeding. # Device or PE-related death during the 48 h after the procedure, procedure-related major bleeding (BARC type 3a or greater), or other procedure-related major adverse events. ** All-cause mortality, or intracranial hemorrhage (ICH), or major bleeding per ISTH definition, or clinical deterioration defined by hemodynamic or respiratory worsening, and/or escalation to a bailout therapy, or ICU admission and ICU length-of-stay during the index hospitalization and following the index procedure. RV, right ventricle. LV, left ventricle. CTA, computed tomography angiography. MAE, major adverse events. FT, Flow Triever.

## 4. Clinical Follow-Up After Transcatheter Treatment

The natural history of a PE may not end at hospital discharge. First, patients with pulmonary embolisms remain at higher risk of PE recurrence and require long-term oral anticoagulation [5]. Secondly, the so-called post-pulmonary embolism syndrome (PPES), reported in up to 40–60% of PE survivors [4], is increasingly being recognized. This is described as new or progressive dyspnea, exercise intolerance, and/or impaired functional or mental status after at least 3 months of appropriate anticoagulation following acute PE, which cannot be explained by other comorbidities [4,42]. Additionally, it has been observed that ∼40% of PE survivors have persistent perfusion defects [4,43,44]. In this context, chronic thromboembolic pulmonary disease (CTEPD) is defined as chronic pulmonary vascular obstruction with normal mean pulmonary artery pressure at rest but with limited exercise tolerance, which is partially attributed to an increased slope of the pulmonary arterial pressure–flow relationship (>3 mmHg/L/min) during exercise or dead space ventilation [45,46,47,48]. Finally, the most severe clinical presentation of PPES is chronic thromboembolic pulmonary hypertension (CTEPH). Although its prevalence is 2–3% in PE survivors and 5–8% in PE survivors with persistent dyspnea [4,43,49,50,51], its consequences may be devastating. The blockage of proximal pulmonary arteries (PA) by fibrotic intravascular material, in combination with a secondary microvasculopathy of vessels <500 μm, leads to increased pulmonary vascular resistance and progressive right heart failure [43].

**Figure 2:**
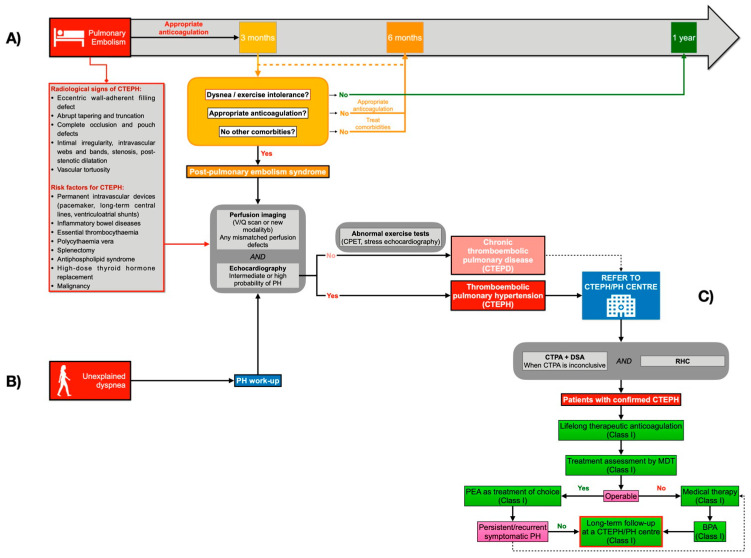
Acute pulmonary embolism diagnostic algorithm for out-of-hospital patient management presenting with acute pulmonary embolism (**A**), dyspnea (**B**) and chronic thromboembolic pulmonary hypertension (CTEPH) (**C**). BPA: balloon pulmonary angioplasty; CTEPH: chronic thromboembolic pulmonary hypertension; CTPA: computed tomography pulmonary angiography; DSA: digital subtraction angiography; PEA: pulmonary endarterectomy; PH: pulmonary hypertension; RHC: right heart catheterization.

It is thought that earlier diagnosis of CTEPH may be relevant for patient outcomes [43,52]. In this review, we propose a follow-up algorithm based on current guidelines and consensus (Figure 2). While current guidelines for acute PE do not recommend routine follow-up of asymptomatic PE patients by imaging methods [43,52], CTEPH should be considered in the following situations: (1) if radiological signs of CTEPH exist on the computed tomography pulmonary angiography (CTPA) performed to diagnose PE and/or if estimated sPAP is >60 mmHg on echocardiogram, (2) when dyspnea or functional limitations persist in the clinical course post-PE, and (3) in asymptomatic patients with risk factors for CTEPH [42,51]. Radiological signs and risk factors for CTEPH are summarized in Figure 2. The most effective tool to exclude PA obstructions is ventilation/perfusion scintigraphy. CTPA with bi-planar reconstruction is broadly used for diagnosis and assessing operability. Digital subtraction angiography is still used to assess treatment options when CTPA is inconclusive [43,52]. Today, the CTEPH treatment algorithms include pulmonary endarterectomy (PEA), balloon pulmonary angioplasty (BPA), and medical therapies to target the mixed anatomical lesions: proximal, distal, and microvasculopathy, respectively [43,52]. For that reason, a multidisciplinary team consisting of cardiothoracic surgeons, pulmonary hypertension specialists, BPA interventionalists, and chest radiologists specializing in CTEPH is key to determining the optimal treatment approach for patients [45,53].

General measures recommended for pulmonary arterial hypertension (PAH) [51] also apply to CTEPH, including supervised exercise training [54,55]. Lifelong therapeutic anticoagulation is recommended for patients with CTEPH, preferably with VKAs, due to the lack of robust evidence for NOACs [5].

Surgical PEA is the treatment of choice for operable patients [45] based on team experience, accessibility of PA lesions, correlation between severity of PH and degree of PA obstructions, and comorbidities. In CTEPH centers, current peri-operative mortality rates are low (2.5%) [56] and long-term outcomes after PEA surgery are excellent regarding hemodynamics (65% decrease in PVR) [57], functional capacity, survival (90% at 3 years), and quality of life [58,59,60,61]. However, up to a third of patients are still considered to be inoperable [62,63,64]. For them, BPA is now a recognized medical intervention, improving hemodynamics (PVR decrease 49–66%), right heart function, and exercise capacity [65,66,67,68,69,70,71,72,73,74,75,76], although evidence about long-term outcomes is still scarce [77]. This is preferable to a phased interventional procedure with a restricted number of dilated PA segments per session. Some of the procedural and post-interventional complications are vascular injury due to wire perforation and lung injury with hemoptysis and/or hypoxia. High-volume CTEPH centers should perform this procedure, as a significant learning curve has been shown [45,52].

Finally, though most of the PAH-targeted therapies have been tested in CTEPH, only riociguat and subcutaneous treprostinil have been approved [78,79]. For these drugs, the level of recommendation for symptomatic patients with inoperable CTEPH or persistent/recurrent PH after PEA are IB and IIaB, respectively. However, the off-label use of drugs approved for PAH may also be considered (IIbB) [52]. Although the role of medical treatments as bridges to interventional and operative treatments is not well studied, current guidelines recommend medical pre-treatment with vasodilators before BPA in patients with a PVR > 4 WU [52,80].

## 5. Future Perspectives and Upcoming Studies

To date, no adequately powered randomized controlled trial has compared transcatheter treatments to the standard of care for pulmonary embolism regarding potential benefits on mortality, recurrence of PE, incidence of post-PE syndromes, and CTEPH, all of which are important sequelae of acute PE. Against this background, several large RCTs are planned (Table 2) and some of them are already recruiting patients. The PE-TRACT trial (NCT05591118) will randomize 500 patients with acute intermediate high-risk PE in an open-label design to either anticoagulation only or transcatheter treatment via CDT or CDMT. The trial is an independent, investigator-initiated phase 3 clinical trial funded by the National Institutes of Health and will assess peak oxygen consumption at 3 months and New York Heart Association functional class at 12 months. In addition, major bleeding at 7 days post-procedure will be assessed. The HI-PEITHO trial (NCT04790370) will enroll 406 patients with acute intermediate–high-risk PE. Participating patients will be randomized 1:1 to either anticoagulation only or anticoagulation plus CDT with the EKOS system. Patients will be followed for 12 months. Outcomes include PE-related death, PE recurrence, cardio–respiratory decompensation, and changes in RV/LV ratio as measured by echocardiography [81]. Moreover, safety will be assessed with ISTH major bleeding events and GUSTO major (moderate and severe) bleeding events. The trial is sponsored by Boston Scientific, the manufacturer and distributor of the EKOS device [81]. The PEERLESS II (NCT06055920) trial will compare large-bore thrombectomy using the FlowTriever device plus anticoagulation against anticoagulation alone (1:1) in 1200 patients with acute intermediate–high-risk PE. The trial aims to assess clinical deterioration (defined by hemodynamic or respiratory worsening), all-cause hospital re-admission, bailout therapy, either after a deterioration or after documented failure to progress after 30 days or at hospital discharge whichever occurred earlier using a win ratio approach. Moreover, dyspnea at 48 h will be assessed. This trial is also sponsored by Inari Medical. In the STRATIFY RCT (NCT04088292), investigators plan to randomly assign 210 patients to one of three treatments: USCDT with 20 mg of alteplase over 6 h plus UFH or LMWH within 12 h of randomization, low-dose systemic thrombolysis with 20 mg of alteplase over 6 h plus UFH or LMWH, or anticoagulation with UFH or LMWH alone. Eligible patients are those with intermediate–high-risk PE, as defined by current ESC guidelines, and a visible thrombus in the main, lobar, or segmental pulmonary artery on CTPA. The primary outcome is the reduction in the Miller obstruction index, with secondary outcomes including bleeding complications, functional parameters, and length of hospital stay, among others. In the ongoing BETULA RCT (NCT03854266), a parallel-design study, low-dose CDT (4 mg or 8 mg alteplase per catheter over 2 h) using the Uni-Fuse system is being compared with heparin alone in 60 patients with intermediate-risk PE and signs of RVD. The primary endpoint is the change in the RV-to-LV ratio 24 h post-procedure. The secondary outcomes include a reduction in thrombus burden after 24 h, 30-day mortality, length of hospital stay, recurrent PE, and lung perfusion. The next-generation Indigo device is currently being evaluated in the observational, single-arm STRIKE PE study. This international, multicenter study will enroll 600 patients with acute PE with a RV-to-LV ratio of ≥0.9 who receive first-line percutaneous treatment with CAVT using the Indigo Aspiration System. The interim results of the first 150 patients (94.7% presented with intermediate-risk PE, and 5.3% with high-risk PE) showed a significant reduction in PAP and RV/LV ratio with a median thrombectomy time of 33.5 min. The composite MAE rate registered was 2.7% [37]. The STORM-PE trial will be the first RCT involving the Indigo Lightning and will soon begin recruitment. This trial aims to enroll 100 patients who will be randomly assigned to either aspiration thrombectomy or anticoagulation alone. The primary endpoint will be the RV-to-LV ratio at 48 h [NCT05684796]. These and other trials (Table 2) will aid in answering important questions: (i) Can percutaneous treatments, besides improving functional aspects, also improve outcomes such as mortality and recurrence of PE? (ii) Are percutaneous treatments as safe as anticoagulation regarding major adverse bleeding events? (iii) Which interventional approach is best suited for which patient? (iv) Are there specific procedural and patient characteristics that may favor one device over the other? (v) What is the optimal timing of the intervention?

## 6. Conclusions

Percutaneous strategies for PE offer a promising alternative for patients who are at high risk or have contraindications to systemic thrombolysis. Current evidence suggests that catheter-based interventions, such as thrombectomy and catheter-directed thrombolysis, provide effective clot removal and swift hemodynamic improvement compared to standard-of-care treatment. However, evidence from large and well-designed randomized clinical trials is needed to confirm the efficacy and safety of these emerging strategies. Finally, optimizing post-procedural management and close follow-up is essential to prevent serious post-PE complications, such as chronic thromboembolic pulmonary hypertension. Early recognition and appropriate management of these complications, using standardized protocols and prompt referral to tertiary care centers, are crucial for improving long-term outcomes and enhancing the quality of life in PE patients.

## Figures and Tables

**Table 2:** Upcoming randomized controlled trials testing transcatheter treatments for pulmonary embolism.

Study	Number of Patients	Cohort	Comparison	Efficacy Outcomes	Safety Outcomes	Sponsor	Strengths	Weaknesses
PE-TRACT (NCT05591118)	500	Intermediate–high-risk PE	CDT or USCDT versus anticoagulation	Peak oxygen consumption, NYHA functional classification	ISTH major bleeding and clinical deterioration	National Insitutes of Health (Bethesda, MD, USA)	Publicly sponsored, comparison to standard of care	Efficacy outcomes mainly functional
HI-PEITHO (NCT04790370)	406 (adaptive design allowing further enrollment)	Intermediate–high-risk PE	USCDT versus anticoagulation	PE-related mortality, PE recurrence, cardiorespiratory decompensation, or collapse	GUSTO and major bleeding per ISTH definition, SAE, all-cause mortality	Boston Scientific (Castle Rock, CO, USA)	Adaptive trial design, comparison to standard of care, well-defined inclusion criteria	Industry-sponsored
PEERLESS II (NCT06055920)	1200	Intermediate–high-risk	Large-bore mechanical thrombectomy versus AC	Win ratio of clinical deterioration, 30-day all-cause readmission, bailout therapy, dyspnea by mMRC	All-cause mortality, all-cause readmission, major bleeding, clinical deterioration	Inari Medical (Irvine, CA, USA)	Large sample size, comparison to standard of care	Industry-sponsored
BETULA (NCT03854266)	60	Intermediate-risk PE and signs of RVD	Low-dose CDT versus anticoagulation	RV/LV ratio, lung perfusion, length of hospital stay	In-hospital mortality, recurrent PE, major and minor bleeding	University of Aarhus (Aarhus Centrum, Denmark)	Publicly sponsored, comparison to standard of care	Small sample size, likely underpowered
STRATIFY (NCT04088292)	210	Intermediate-risk PE	USCDT versus low-dose systemic thrombolysis versus anticoagulation	Reduction in Miller score	TIMI bleeding, length of hospital stay, subjective dyspnea, mortality	Rigshospitalet, Copenhagen, Denmark	Publicly sponsored, three arms, comparison to standard of care	
STORM PE (NCT05684796)	100	Intermediate–high-risk	Medium-bore mechanical thrombectomy versus AC	RV/LV ratio, functional outcomes, and quality of life assessments at 90 days	Clinical deterioration, mortality, symptomatic recurrent PE, or major bleeding within 7 days	Penumbra Inc. (Livermore, CA, USA)	Comparison to standard of care	Industry sponsored
